# Outbreaks of *Mycobacterium tuberculosis* MDR strains differentially induce neutrophil respiratory burst involving lipid rafts, p38 MAPK and Syk

**DOI:** 10.1186/1471-2334-14-262

**Published:** 2014-05-16

**Authors:** María Mercedes Romero, Juan Ignacio Basile, Beatriz López, Viviana Ritacco, Lucía Barrera, María del Carmen Sasiain, Mercedes Alemán

**Affiliations:** 1 Inmunologia de enfermedades respiratorias, IMEX-CONTICET-ANM, Buenos Aires, Argentina; 2 Servicio de Micobacterias, Instituto Nacional de Enfermedades Infecciosas, ANLIS “Carlos G. Malbran”, Buenos Aires, Argentina

## Abstract

**Background:**

Neutrophils (PMN) are the first cells to infiltrate the lung after infection, and they play a significant protective role in the elimination of pathogen, by releasing preformed oxidants and proteolytic enzymes from granules and generating ROS, thus limiting inflammation by succumbing to apoptosis. In a previous study, we found marked differences in ROS-induced apoptosis between two *Mycobacterium tuberculosis (Mtb)* strains, M and Ra, representative of widespread *Mtb* families in South America, i.e. Haarlem and Latin-American Mediterranean (LAM), being strain M able to generate further drug resistance and to disseminate aggressively.

**Methods:**

In this study we evaluate the nature of bacteria-PMN interaction by assessing ROS production, apoptosis, lipid raft coalescence, and phagocytosis induced by *Mtb* strains.

**Results:**

Dectin-1 and TLR2 participate in *Mtb*-induced ROS generation and apoptosis in PMN involving p38 MAPK and Syk activation with the participation of a TLR2-dependent coalescence of lipid rafts. Further, ROS production occurs during the phagocytosis of non-opsonized bacteria and involves α-glucans on the capsule. In contrast, strain M lacks the ability to induce ROS because of: 1) a reduced phagocytosis and 2) a failure in coalescence of lipid raft.

**Conclusions:**

The differences in wall composition could explain the success of some strains which stay unnoticed by the host through inhibition of apoptosis and ROS but making possible its replication inside PMN as a potential evasion mechanism. Innate immune responses elicited by *Mtb* strain-to-strain variations need to be considered in TB vaccine development.

## Background

Tuberculosis (TB) remains a major global health problem caused by inhalation of aerosols containing *Mycobacterium tuberculosis* (*Mtb*). Ten million new TB cases and 2 million deaths are estimated to occur each year, more than any time in history [1]. The risk of disease is also increased by emergence of acquired immune deficiency syndrome and multidrug-resistant (MDR) *Mtb* strains [1]. Whether bacterial lineage influences on the development of TB disease is uncertain, although there is long-standing evidence that some *Mtb* strains are more virulent than others and vary in their ability to cause disease in humans [2].

*Mtb* has evolved successful strategies to invade and persist within host cells and these interactions appear to involve surface polysaccharides and glycolipids present in the *Mtb* surface [3,4]. The capsule is the outermost compartment of the bacteria envelope [5,6] and is composed of carbohydrates and proteins with only small amounts of species- or type-specific lipids [7,8]. *Mtb* capsular carbohydrates are absent in some other taxa and mediate specific interactions with the host [9]. The major carbohydrate constituents from *Mtb* surface are α-glucans, which represent up to 80% of the extracellular polysaccharides. These α-glucans are composed of a 4-α-DGlc-1 core branched at position 6 every five or six residues by 4-α-D-Glc-1 oligoglucosides [7,8,10].

Recognition of mycobacterial components by Toll-like receptors (TLRs) [11] is a key step in initiating innate immune responses upon mycobacterial infection. In this context, it has been demonstrated that TLR2 stimulation enhances production of reactive oxygen species (ROS) that is accompanied by sustained phosphorylation of p38 mitogen-activated protein kinase (p38 MAPK), suggesting an essential role of ROS in TLR2 signaling pathways [12]. Moreover, several C-type lectin receptors also participate in the recognition of mycobacteria. Among them, dectin-1 is a phagocytic receptor for fungal wall-derived β-glucans that is expressed on monocytes (Mo), macrophages (MØ), neutrophils (PMN), dendritic cells (DC) and Langerhans cells [13]. Dectin-1 was described to induce ROS production in MØ [14] involving the spleen tyrosine kinase (Syk) activation [15]. The role of dectin-1 in mycobacterial infections has begun to be studied recently, and its role in the functionality of PMN has not been investigated so far.

The most successful *Mtb* genotypes in South America belong to three Euro-American families, i.e., Latin-American Mediterranean (LAM), ill-defined T, and Haarlem [16]. In Argentina, the only South America country where large MDR-TB epidemics have been documented [17], there are two main mycobacterial clusters, strain M belonging to the Haarlem family, and strain Ra belonging to LAM family [18]. In particular, strain M disseminated aggressively building up further drug resistance without impairing its ability to spread and persist in the community.

Although MØ are the major target of *Mtb* infection, how the innate immunity mediates host defense against mycobacteria has long remained poorly understood. One of the first events in the pathogenesis of the disease is the influx of PMN to the lung. Being the most commonly infected phagocytes in human TB [19], PMN play a significant protective role in the elimination of invading pathogens through the generation of ROS [20] and the release of preformed oxidants and proteolytic enzymes from granules [21], thereby contributing to the control of *Mtb* infection [22]. In addition, PMN apoptosis can be triggered with non-opsonized *Mtb*[23] involving TLR2 and p38 pathway [24,25] and oxidative processes, which have been implicated in mycobacterial pathogenesis [26]. The strict dependence of *Mtb*-induced apoptosis on ROS production [23] suggests an important host defense mechanism for removal of the infected PMN from the site of infection which, in turn, can limit inflammation [27].

In a previous paper we showed that clinical strains from LAM and Haarlem families differ in their ability to induce ROS and apoptosis in PMN, a mechanism that would allow immune evasion and survival of both bacteria and PMN [28]. Herein, we investigated the mechanisms that govern *Mtb*-induced ROS production in PMN and the role of the major carbohydrate constituent of *Mtb* capsule, α-glucan.

## Methods

### Ethics compliance

All procedures were performed in compliance with institutional guidelines and the relevant institutional committee (Ethics Committee of the National Academy of Medicine in Buenos Aires) approved our research. All healthy volunteers signed a written informed consent.

### *Mtb* clinical isolates

*Mtb* clinical isolates were obtained from sputum culture positive patients. The isolates had been previously submitted to drug susceptibility testing and genotyping by IS*6110* DNA fingerprinting and spoligotyping using standardized protocols [18]. Two multidrug-resistant strains (MDR) were employed in this study: Ra 11608 and M 6548 belonging to LAM and Haarlem families respectively [28]. The isolates belonged to the collection kept at the Reference Laboratory for Mycobacteria, Instituto Nacional de Enfermedades Infecciosas ANLIS “Carlos G. Malbran” in Buenos Aires, Argentina. All strains were grown in Middlebrook 7H9 broth (Difco Laboratories, Detroit MI, USA) at 37°C in 5% CO_2_ until log phase. Mycobacteria were harvested, washed three times and suspended in phosphate buffered saline (PBS) free of pyrogen. Bacteria were killed by gamma irradiation (2.4 megaRads) and suspended in PBS at an OD_600_ nm of 1 (~10^8^ bacteria/ml) and stored at −20°C until their use. When indicated, bacteria were heat killed at 121°C for 30 min. (ht *Mtb*).

### Antibodies and reagents

Oxidase inhibitor, diphenyleneiodonium (DPI) was provided by Cayman Chemical (Michigan, USA), the specific inhibitor of p38 (SB203580) and the Syk inhibitor Piceatannol (Pic) were purchased from Calbiochem-Behring (La Jolla, CA, USA). The FITC-conjugated B subunit of cholerae toxin (CTB), Annexin V-FITC, the lipid raft inhibitor β-Metil-ciclodextrin (MβC), the β-glucan Laminarin, PMA, Pam3Cys, cytochalasin D, orthovanadate and amyloglucosidase (A3514, EC 3.2.1.3) were purchased from (Sigma Chemical Co., St. Louis, Mo, USA) and dihydrorhodamine 123 (DHR) was purchased from Invitrogen.

Mouse antibodies (Abs) against phospho-(Thr180/Tyr182)-p38 were purchased from Santa Cruz Biotechnology (Santa Cruz, CA, USA). Rabbit antihuman p-(Tyr525/526)-Syk was purchased by Cell Signaling Technology (Cell Signaling Technology, Inc., Danvers, MA), donkey anti-mouse-IgG-FITC, Mouse antihuman-TLR2-FITC (clone TL2.1) and blocking antibodies against human CD11b and TLR2 (clone TL2.1) were purchased by Biolegend (Biolegend, San Diego, CA). Mouse antihuman-dectin-1-PE (2A11) and mouse antihuman-dectin-1 (clone 259931) were purchased by R&D (R&D Systems inc. Minneapolis USA) as well as their corresponding isotype.

### Amyloglucosidase digestion of the α-glucan on *Mtb* capsule

Clinical strains Ra and M were treated with the enzyme α-amyloglucosidase (Sigma Chemical Co.). To remove α-glucans from the capsule, bacteria were centrifuged at 100 g and suspended in 1 ml of α-amyloglucosidase (500 U/ml) prepared in sodium acetate buffer 0.05 M and 0.1 M NaCl, pH 4.5. As a control, the bacteria were also suspended in enzyme-free buffer. Bacteria suspensions were incubated at 37°C for 18 hr and then centrifuged at 100 g. The supernatants were discarded, and the pellets were washed with PBS. Thereafter, treated bacteria (*Mtb* e) were suspended in 1 ml of PBS and stored at −20°C until their use.

### PMN purification and culture

PMN were isolated from heparinized venous blood from healthy donors by Ficoll-Hypaque gradient centrifugation for 40 min. at 350 g and 4°C [29] followed by 20 min. sedimentation in 3% dextran (Sigma). The PMN-rich supernatant was collected and residual red blood cells were removed by hypotonic lysis. The cells were washed immediately and suspended at 3 × 10^6^ cells/ml in RPMI-1640 medium (Gibco, NY, USA) supplemented with 1% heat-inactivated Fetal Calf Serum (FCS) (Gibco) and 50 μg/ml gentamycin (complete media, CM). The viability was consistently >95% as determined by trypan blue dye exclusion. The purity of the PMN preparation was up to 95% as assessed by morphological examination by staining with Wright-Giemsa and by FACS light scatter pattern. Cultures were performed by incubating 1 ml of a PMN suspension (3 × 10^6^ cell) in Falcon 2063 tubes stimulated at different *Mtb*:PMN ratios as indicated (1:2; 2:1; 5:1 to induce cell signaling, 10:1 to see phagocytosis).

### Flow cytometric analysis of cells surface phenotype

Cell surface expression of TLR2 and dectin-1 in recently isolated or 30, 60 and 180 min-cultured PMN was evaluated by direct immunofluorescence using saturating concentrations of monoclonal mouse antihuman TLR2-PE- and dectin-1-FITC-conjugated antibodies. Briefly, 5 × 10^5^ cells were incubated with the antibody for 20 min on ice, and, after 20-min incubation on ice in the dark, cells were washed and fixed in 500 μl of 1% paraformaldehyde. Thereafter, 10,000 events were collected in linear mode for forward scatter (FSC) and side scatter (SSC), and log amplification for FL-1 and FL-2, using a FACScan (Becton-Dickinson Immunocytometry Systems, San Jose, CA). Analysis was performed using the FACS express software (De Novo Software) and isotype matched controls were used to determine auto-fluorescence and nonspecific staining. Results were shown as percentages of positive cells and as mean fluorescence intensity (MFI).

For intracellular staining of cytoplasmic proteins, PMN were permeated by using a Fix and Perm kit (Caltag, Burlingame, CA, USA). Briefly, to detect the phosphorylated form of p38, 3 × 10^6^ PMN were incubated with different *Mtb* strains at 1:2 *Mtb*:PMN ratio for 30 min., and thereafter cells were washed and resuspended in 100 μL solution A (fixation) for 15 min at room temperature. After washing with PBS containing 1% Na azide and 5% FCS, cells were resuspended in solution B (permeabilization) and mouse antihuman p-p38 IgM anti-p-p38-FITC revealed with rabbit anti-mouse-IgG-FITC antibodies. To detect the phosphorylated form of Syk, PMNs were incubated with different *Mtb* strains at 1:2 *Mtb*:PMN ratio for 30 min., in the presence of ortovanadate in order to avoid phosphatases activation. After washing with PBS containing 1% Na azide and 5% FCS, cells were suspended in solution B (permeabilization) and mouse antihuman anti-p-Syk revealed with donkey anti-mouse-IgG-FITC antibodies. After 20-min incubation on ice in the dark, cells were washed, resuspended in isoflow and acquired in a flow cytometry and analyzed as previously described.

### Oxidative Burst Assay

Intracellular ROS were measured by 123Dihydrorhodamine assay (DHR). DHR was dissolved in DMSO (Sigma) at 20 μg/ml and stored in aliquots at −70°C until used. Briefly, 5 × 10^5^ PMN were incubated with 100 μl DHR (5 μg/ml) for 15 min at 37°C and afterward, *Mtb* (1 × 10^6^ bacteria/ml) were added to the culture at 1:2 *Mtb*:PMN ratio for additional 90 min. When indicated, PMN were pre-incubated with the free glucan laminarin (10 μg/mL) [30] or with anti-dectin-1 or anti-TLR2 blocking antibodies. Alternatively, the oxidase inhibitor, DPI (0.1 mM), cytochalasin D (1 μg/mL) as well as the inhibitors of Syk (piceatannol, 15 mM) and p38 (SB20358, 20 μM) where used before *Mtb* challenge. Afterward PMN were washed, suspended in isoflow and thereafter, 10,000 events were collected in linear mode FSC and SSC and log amplification for FL-1, in a flow cytometer as mentioned above. When indicated, ROS production was also induced by DHR-labeled *Mtb* (^DHR^*Mtb*). Briefly, *Mtb* were incubated with 5 μg/ml DHR for 30 min at 37°C followed by an extensive washing to remove free DHR and suspended in RPMI 1640 plus 10% FCS. Thereafter, 5 × 10^5^ PMN were incubated with ^DHR^*Mtb* strains at a 10:1 ratio for 90 min at 37°C, washed and centrifuged at 100 *g* evaluated immediately in a flow cytometer as previously described.

### Detection of Apoptosis Using Annexin-FITC

The percentage of apoptotic PMN was assessed based on the Annexin V-FITC (Sigma) protein-binding assay. Briefly, 5 μL of Annexin V-FITC (10 μg/mL) and 5 μL of propidium iodide (PI) (250 mg/mL) (Sigma) were added to 1.2 × 10^6^ cells in 500 μL of binding buffer and incubated for 15 min at room temperature in the dark. When indicated, PMN were pre-incubated with the free glucan laminarin or with anti-dectin or anti-TLR2 blocking antibodies. Alternatively, the oxidase inhibitor, DPI, as well as Syk or p38 inhibitors, piceatannol and SB20358 (20 μM) respectively, where used before *Mtb* challenge. Cells were washed, resuspended in binding buffer acquired in a flow cytometry, as previously described [24]. AV*-*/PI*-* cell population was regarded as alive, AV+/PI- was considered as an early apoptotic population, and the AV+/PI + population represented late stage apoptotic cells. The sum of the last two populations were considered, and expressed in the results. Inhibitors used in these experiments did not affect cell viability [24].

### Phagocytosis assay

Phagocytosis of *Mtb* was evaluated by the method described by Busseto et al. [31]. Briefly, 3 × 10^6^/ml PMN were suspended in media with 10% FCS and incubated in a shaking water bath with opsonized or unopsonized FITC-labeled *Mtb* strains for 2 hr at 37°C at a 10:1 ratio *Mtb*:PMN. To stop phagocytosis, aliquots of the incubation mixtures were withdrawn within an equal volume of Trypan Blue (250 μg/ml) dissolved in ice-cold citrate buffer (0.1 M, pH 4.0). After 1-min incubation in ice, the samples were analyzed by flow cytometry. Each sample was collected for 30 seconds at the slowest flow rate to minimize the coincidental appearance of free bacteria and PMN in the laser beam. Opsonization of *Mtb* was performed by employing sera from PPD- individuals in order to avoid the effect mediated by specific antibodies. When indicated, bacteria where enzimatically treated to eliminate α-glucans from capsule, as described above. To evaluate CD11b-mediated phagocytosis, cells were previously incubated with non relevant IgG1 or with anti-CD11b blocking antibodies, for 30 min at 37°C before incubation with *Mtb* strains. The data were then analyzed by using CellQuest software from Becton Dickinson. The mean number of ingested bacteria per PMN was assessed (i.e., both attached and internalized particles).

### IL-8 detection in PMN

Enzyme-linked immunosorbent assays (ELISA) were performed to evaluate IL-8 secretion by PMN in response to *Mtb* strains. To that, supernatants from 18 hr-cultured PMN with different stimuli, were analyzed for cytokine content using sandwich ELISA (BD Pharmingen) performed as recommended by the manufacturers.

### Lipid rafts detection

#### Flow cytometry

The formation of lipid rafts was determined by the increased expression of the molecule GM1 in the membrane of PMN, which binds B subunit of cholera toxin conjugated with FITC (CTB). For this, 5 × 10^5^ PMN were incubated in RPMI 1640 10% FBS against different clinical strains at 2:1 *Mtb*:PMN ratio, for 30, 60 or 120 min. at 37°C and 5% CO2. Then the cells were washed with PBS and labeled with CTB (5 μg/ml) for 30 min on ice. When indicated PMN were treated with DPI to inhibit ROS production previous to *Mtb* stimulation, or were stimulated with *Mtb* subjected to an enzymatic treatment to eliminate α-glucans from the cell wall. Finally the cells were washed with PBS, suspended in isoflow and immediately detected by flow cytometry broadcast FL-1 channel.

#### Confocal microscopy

After cultured and incubated with CTB, the PMN were labeled with Propidium (250 ug/ml) to visualize the nucleus, fixed in a solution of 4% PFA and centrifuged. The formation of lipid rafts was determined by the presence of clusters of CTB expression by confocal microscopy (Olympus) and was analyzed using the program FV10-ASW 1.7 Viewer.

### Statistics

The statistical analysis of the data was performed using one-way ANOVA followed by Tukey’s multiple comparison tests to compare more than two groups followed by Wilcoxon test to compare two groups. The significance adopted was p < 0.05. The graphical representation of the values is given by mean ± SEM.

## Results

### Dectin-1 and TLR2 participate in *Mtb-*induced ROS generation and apoptosis in PMN

**Figure 1:**
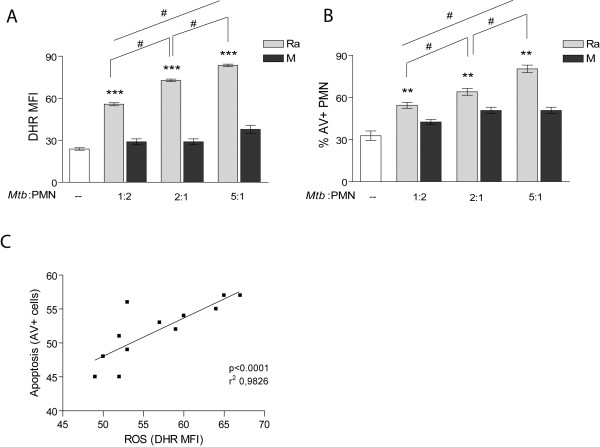
**ROS induced by strain Ra is dose dependent and correlates with apoptosis. (A)** PMN were pretreated with DHR and incubated with different relations of *Mtb*:PMN (1:2, 2:1 and 5:1). Thereafter, the emission of oxidized DHR was evaluated by flow cytometry as a measure of oxidative burst. The results are expressed as mean ± SE (n = 16); control (−−) vs. all *Mtb*:PMN ratios ***p < 0.001; 1:2 vs. 2:1; 1:2: vs. 5:1 and 2:1 vs. 5:1 *Mtb*:PMN ratio: ^#^p < 0.05 **(B)** Apoptosis was measured in 18-hr cultured PMN with or without the presence of clinical strains as described in **(A)**. Results are expressed as the percentage of AV-FITC positive cells (% PMN AV + ± SE) (n = 16); control (−−) vs. all *Mtb*:PMN ratios: **p < 0.001; 1:2 vs. 2:1; 1:2: vs. 5:1 and 2:1 vs. 5:1 *Mtb*:PMN ratio: ^#^p < 0.05; **C)** correlation between ROS generation and apoptosis of PMN (p < 0.0001, r^2^: 0.9826).

Clinical strains Ra and M were chosen because of the disparity in their ability to induce apoptosis and ROS production in human PMN that were not due to drug resistance [28]. As it is shown in Figure 1A and B, whereas strain M was unable to induce ROS or apoptosis in PMN, Ra induced high level of ROS in a dose dependent manner, which correlated with apoptosis rate (Figure 1C). The fact that ROS is induced at a low ratio of non-opsonized *Mtb* to PMN (1:2), would be important during the early phase of infection when serum opsonins are limited. Therefore we choose the lower ratio in which differences in ROS and apoptosis between strains were significant.

**Figure 2:**
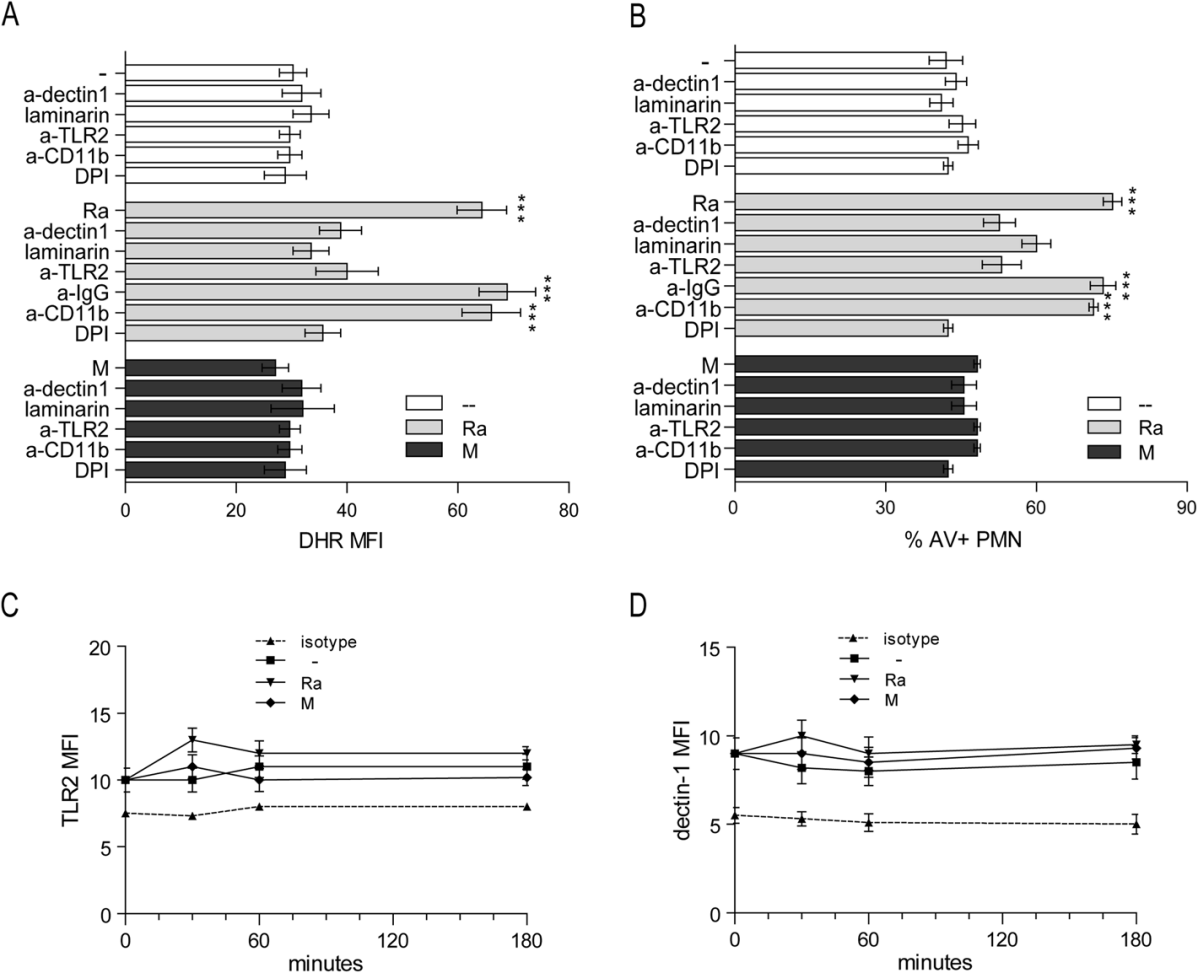
**dectin-1 and TLR2 participate in *Mtb-*induced ROS and apoptosis in PMN. A)** PMN were pretreated with DHR and incubated with or without neutralizing anti-TLR2, anti-dectin-1, anti-CD11b, irrelevant IgG antibodies or the β-glucan laminarin, and then stimulated with Ra or M for 90 min at 1:2 ratio *Mtb*:PMN. Thereafter, the emission of oxidized DHR was evaluated by flow cytometry. Results are expressed as mean ± SE (n = 12); control (−−) vs. Ra, Ra + IgG and Ra + anti-CD11b: ***p < 0.001 **(B)** Apoptosis was measured in 18-hr cultured PMN with or without clinical strains, under the same conditions described in **(A)** and evaluated by flow cytometry. Results are expressed as the percentage of AV-FITC positive cells (% PMN AV + ± SE) (n = 20); control (−−) vs. Ra, Ra + IgG and Ra + anti-CD11b: ***p < 0.001. **(C)** TLR2 and **(D)** dectin-1 expression was measured at the surface of the PMN after 30, 60 or 180 minutes culture, in the presence of *Mtb* (1:2 *Mtb*:PMN ratio) (n = 16) and evaluated by flow cytometry.

Given that TLR2 has been shown to be involved in apoptosis induced by H37Rv in PMN [25] and in turn it cooperates with dectin-1 in the recognition of non virulent mycobacteria such as *M. smegmatis* in MØ [26], we studied the implication of TLR2 and dectin-1 in the generation of ROS induced by clinical isolates. We found that Ra-induced ROS production was significantly reduced by blocking dectin-1 with specific antibodies or with laminarin, a soluble β-glucan. Notably, blockade of TLR2 reduced the level of ROS to baseline, suggesting cooperative participation with dectin-1 to activate signaling (Figure 2A). As previously reported, the CD11b was not involved in the generation of ROS by *Mtb*[32], since CD11b blocking did not affect ROS production. Figure 2B reflects a clear dependence between Ra-induced apoptosis and ROS production that has been evidenced by the effect of NADPH oxidase inhibitor diphenyleneiodium (DPI). In clear contrast, none of the treatments modified the effect of the strain M on apoptosis or ROS production.

To rule out that the increased production of ROS was due to an up-regulation of dectin-1 and/or TLR2 on PMN surface, the expression of these receptors was measured by flow cytometry on freshly isolated or cultured PMN with media alone or exposed to *Mtb* strains for 30, 60 or 180 min. As is shown in Figure 2C and D, neither strains Ra nor M induced a change on TLR2 or dectin-1 expression in PMN, so we can discard a receptor modulation as a cause of the increased ROS production induced by Ra.

### *Mtb*-induced ROS production involves p38 MAPK and Syk activation

**Figure 3:**
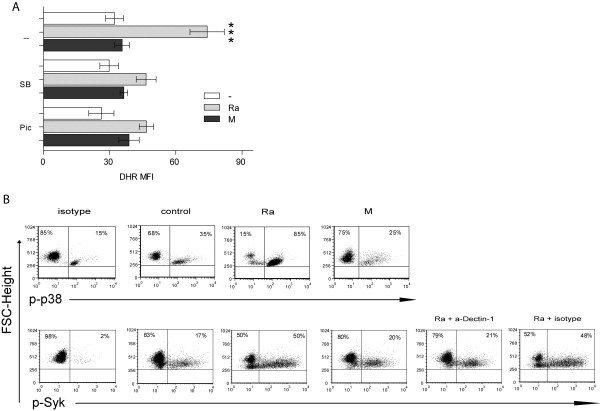
**Role of p-38-MAPK and Syk in *Mtb*-induced ROS production by PMN. (A)** PMN were pretreated with DHR and incubated with specific p38 MAPK and Syk inhibitors, SB203580 and piceatannol, respectively (SB and Pic) for 30 min. previous to bacteria challenge (1:2 *Mtb*:PMN ratio for 90 minutes). Thereafter, the emission of oxidized DHR was evaluated by flow cytometry. Results are expressed as mean ± SE (n = 20); Ra alone vs. all conditions: ***p < 0.001. **(B)** Percentage of cells that express the activated form of cytoplasmic p38 MAPK and Syk. PMN were incubated with Ra or M strains with neutralizing anti-dectin-1 or corresponding isotype. A representative experiment of 10 done is depicted.

Considering that dectin-1 and TLR2 participate in ROS production induced by Ra, we evaluated the role of Syk and p38 signaling cascade downstream of these receptors. As shown in Figure 3A, the Ra-induced ROS production was remarkably reduced by the specific inhibitor of Syk activation, piceatannol (Pic). Moreover, SB203580, the specific inhibitor of p38 MAPK activation also reduced ROS to baseline, suggesting that both molecules may participate in ROS generation.Then, in order to assess whether the observed differences in the ability to induce ROS by strains Ra and M could be due to a differential capacity to activate p38 MAPK and Syk, the expression of their activated form in the cytoplasm was examined. As shown in Figure 3B, Ra strain significantly induced activation of p38 MAPK and Syk, whereas M was not able to induce activation over baseline. Furthermore, Ra-induced Syk activation was dependent on dectin-1 receptor because it was significantly inhibited by the blockade of dectin-1.

### *Mtb* strains differentially induce GM1 expression in PMN

**Figure 4:**
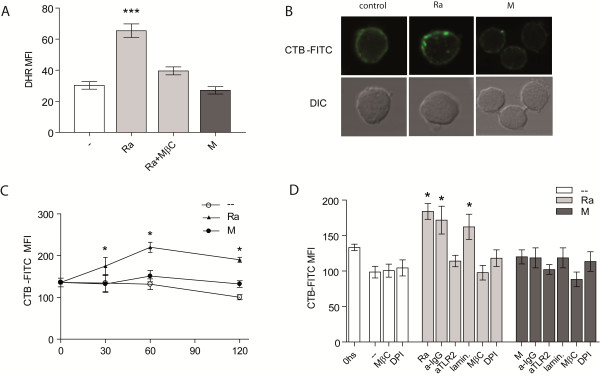
**Ra strain induces GM1 expression in PMN surface. (A)** PMN treated with DHR were incubated with the lipid rafts inhibitor MβC, before challenge with 1:2 *Mtb*:PMN. Thereafter, the emission of oxidized DHR was evaluated by flow cytometry. Results are expressed as mean ± SE (n = 12); Ra vs. control (−−), Ra + MβC or M: ***p < 0.001 **(B)** PMN were cultured with bacteria and incubated with CTB-FITC and thereafter, lipid rafts were determined by the presence of clusters of GM1 expression by confocal microscopy. Propidium yodide (250 μg/ml) was used to visualize the nucleus. One of 4 experiments is depicted **(C)** PMN were cultured with a 2:1 *Mtb*:PMN ratio for 30, 60 o 120 min. Thereafter, cells were labeled with CTB-FITC (5 μg/ml) and fluorescence evaluated by flow cytometry. Results are expressed as mean ± SE (n = 7); control (−−) vs. Ra: *p < 0.05. **(D)** PMN were incubated with the ROS inhibitor DPI, MβC, laminarin, anti-TLR2 or irrelevant antibody for 30 min. at 37°C before challenge with Ra or M at 2:1 *Mtb*:PMN ratio. Thereafter, cells were labeled as described in **(C)**. Results are expressed as mean ± SE (n = 7); control (−−) vs. Ra, laminarin or irrelevant: *p < 0.05.

Lipid rafts are membrane microdomains enriched in cholesterol and glycosphingolipids that have been implicated in signal transduction and immune responses [33]. Components of the NADPH oxidase do indeed localize in lipid microdomains indicating that lipid rafts mediate the efficiency of oxidase coupling to a receptor in PMN membrane [34]. The role of lipid rafts in *Mtb*-induced ROS production was assessed by employing Methyl-β-cyclodextrin (MβC), which disrupts lipid rafts by removing cholesterol from membranes. As can be seen in Figure 4A, MβC caused a significant decrease in Ra-induced ROS production, indicating that a proper coalescence of lipid rafts is a requisite in ROS generation induced by this strain.

Therefore, expression of the GM1 molecule was determined in the membrane of PMN, through binding of cholera toxin B subunit conjugated with FITC (CTB-FITC) [35]. Ratio 2:1 *Mtb*:PMN was used for CTB measure, to see clearly the differences in the image, whereas M did not induce ROS (see Figure 1A). A uniform distribution of GM1 can be observed in untreated PMN or in PMN treated with strain M, while GM1 was clustered into patches in Ra-stimulated PMN (Figure 4B). Thereafter, GM1 expression was measured by flow cytometry, considering that higher GM1 clustering is shown as higher fluorescence intensity [36]. Noticeably, strain Ra induced an increase in GM1 expression that reached a peak at 60 min-culture, while it was constant over time in the control without stimuli or in M-stimulated PMN (Figure 4C).

### *Mtb*-induced GM1 expression in PMN is dependent on TLR2

It has been described that TLR2 agonists of *Mtb* induce coalescence of lipid rafts, translocation of TLR2 to lipid rafts, and ROS production [37]. As showed above, TLR2 participates in Ra-induced ROS production, thus we investigated the role of TLR2 in the up-regulation of GM1 expression induced by our strains. As shown in Figure 4D, whereas laminarin had not effect, the blockage of TLR2 abrogated the increase of GM1 expression suggesting that only TLR2 and not dectin-1 is involved in lipid raft coalescence.On the other hand, pre-treatment with DPI hampered the GM1 expression in PMN membrane, suggesting that ROS are necessary for the aggregation of raft domains (Figure 4D). Therefore, it seems that while lipid rafts participate in ROS production induced by strain Ra, in turn ROS is necessary for lipid raft coalescence.

### Phagocytosis of nonopsonized bacteria involves α-glucans on cell wall and is strain dependent

**Figure 5:**
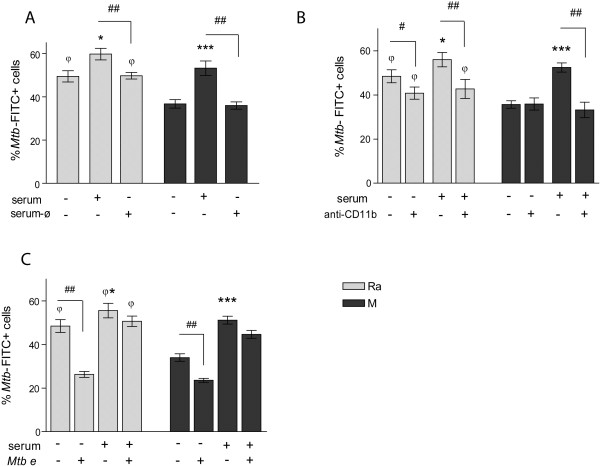
**Non-opsonic phagocytosis is strain dependent and involves α-glucans on *Mtb. *(A)** PMN were incubated with FITC labeled-*Mtb* opsonized (serum) with intact or heated (ø) PPD negative serum (10:1 *Mtb*:PMN). Results are expressed as% of PMN that phagocytosed *Mtb* (% PMN *Mtb* FITC ± SE); Ra vs. Ra + serum: *p < 0.05; M vs. M + serum: ***p < 0.001; Ra vs. M: ^φ^p < 0.05; R + serum vs. R + serum-ø: ^##^p < 0.01; M + serum vs. M + serum-ø: ^##^p < 0.01 **(B)** PMN were incubated with blocking anti-CD11b antibodies prior to incubation with FITC-*Mtb* opsonized or not. Results are expressed as% of PMN that phagocytosed *Mtb* (% PMN *Mtb* FITC ± SE); Ra vs. Ra + serum: *p < 0.05; M vs. M + serum: ***p < 0.001; Ra vs. Ra + aCD11b: ^#^p < 0.05; Ra + serum vs. Ra + serum + aCD11b: ^##^p < 0.05; M + serum vs. M + serum + aCD11b: ^##^p < 0.05; Ra, Ra + aCD11b and Ra + serum + aCD11b vs. M, M + aCD11b and M + serum + aCD11b: ^φ^p < 0.02 **(C)** PMN were incubated with opsonized or non-opsonized FITC-*Mtb* that were treated (*Mtb e*) or not with α-glycosidase. Results are expressed as% of PMN that phagocytosed *Mtb* (% PMN *Mtb* FITC ± SE); Ra vs. Ra + serum: *p < 0.05; M vs. M + serum: ***p < 0.001; Ra vs. Ra e and M vs. M e: ^##^p < 0.01; Ra, Ra + serum and Ra e + serum vs. M, M e and M e + serum: ^φ^p < 0.05.

We have previously reported a more efficient entry of Ra into PMN compared to strain M [28]. Therefore, in order to evaluate the nature of these differences, a phagocytosis assay was performed with both non-opsonized and opsonized *Mtb* using a high ratio *Mtb*:PMN (10:1) due to a limitation of the assay, just to see fluorochrome emission. As shown in Figure 5A, bacterial opsonization improves phagocytosis of both strains M and Ra, which was prevented by exposure of serum to 56°C for 30 minutes (denaturing of complement factors, serum-ø) suggesting that phagocytosis mediated by complement factors is similar in both strains, whereas when non-opsonized, M was less efficient than Ra to enter PMN.

Among a variety of *Mtb* receptors in the membrane of PMN, CD11b is one of the main receptor of phagocytosis which binds complement factor C3bi, as well as non-opsonized bacteria at a different site from C3bi binding site [38]. As shown in Figure 5B, complement-mediated phagocytosis of both strains was reduced by CD11b blockade whereas it only affected the non-opsonic phagocytosis of Ra. Although a differential binding to CD11b was not evaluated, these results suggest that differences between strains arise in the lectin like domain. However, even with the blockade, phagocytosis of unopsonized M remained significantly lower than Ra so that we cannot rule out differences at the binding to other receptors.

It has been described that *Mtb* interacts with CD11b, through capsular polysaccharides, which may be dependent on the strain. Moreover, the binding range of the CD11b lectin site can be extended to include a glycogen-like α-1,4-glucan from the *Mtb* capsule indicating that outer capsular polysaccharides mediate the non-opsonic binding to CD11b [39]. In order to evaluate whether α-glucan of the capsule participates in the entrance of *Mtb*, clinical strains were treated with 1,4-α-glycosidase to remove α-glucans, and the phagocytosis of opsonized and non-opsonized bacteria was compared with intact bacteria. As can be seen in Figure 5C, elimination of α-glucans dramatically reduced phagocytosis of non-opsonized bacteria whereas complement-mediated phagocytosis was not significantly altered, suggesting that capsular polysaccharides participate in the phagocytosis of non-opsonized *Mtb* and are strain dependent.

### ROS production in PMN involves phagocytosis and α-glucans on *Mtb*

**Figure 6:**
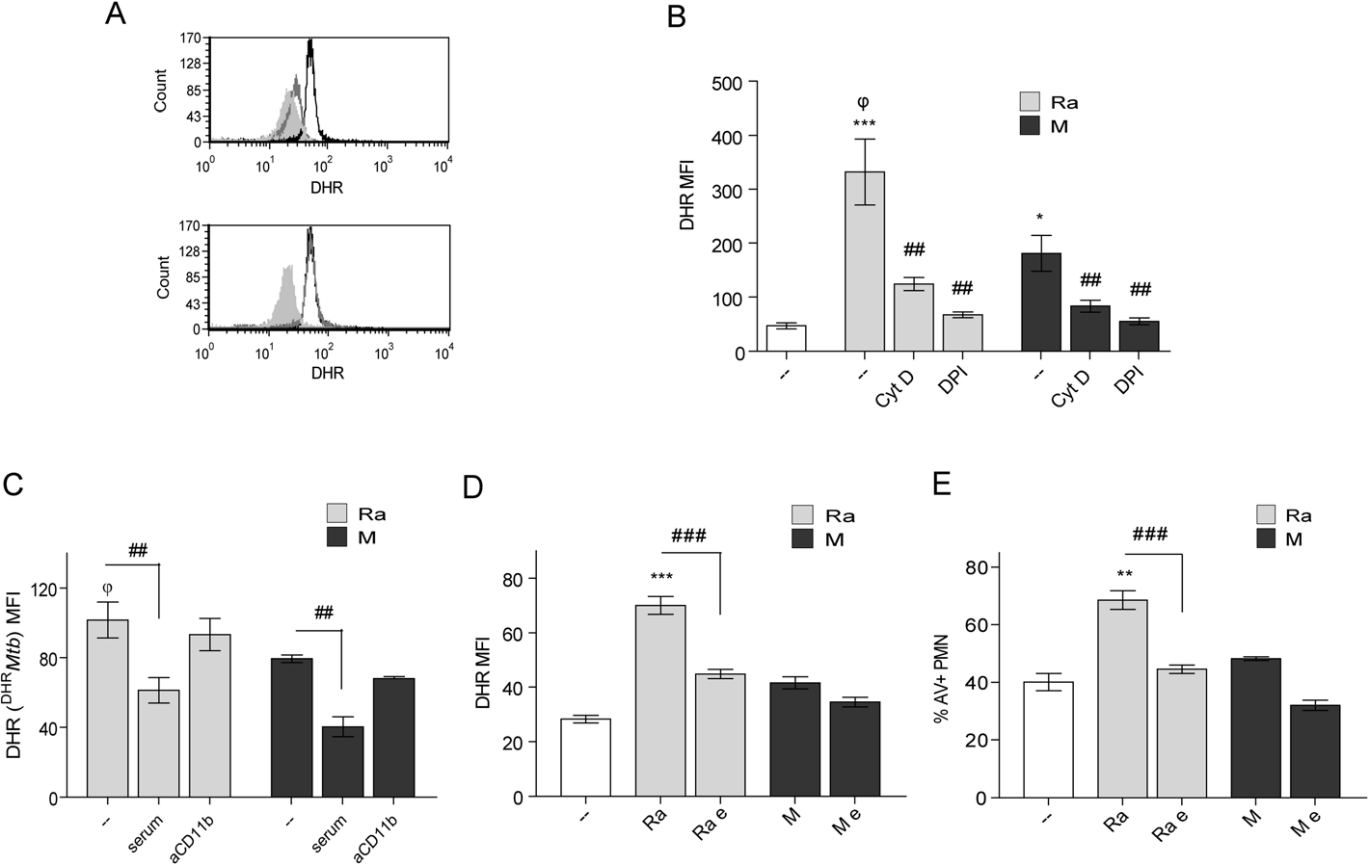
**α-glucans and non-opsonized phagocytosis are involved in ROS production. (A)** PMN were pretreated with DHR and incubated with 1:2 *Mtb*:PMN ratio for 90 min (black line), pre-incubated with cytochalasin D (gray line) or, PMN without treatment (filled gray). Alternatively, PMN were pre-treated with anti-CD11b, second histogram (gray line). Representative out of 6 experiments are depicted **(B)** PMN pretreated with DHR were incubated with cytochalasin D or DPI prior to incubation with 10:1 *Mtb*:PMN ratio for 90 min. Results are expressed as a mean ± SE (n = 20); control (−−) vs. Ra ***p < 0.001; control (−−) vs. M *p < 0.05; Ra vs. M ^φ^p < 0.01; Ra vs. Ra + cytD or Ra + DPI: ^##^p < 0.01; M vs. M + cytD or M + DPI ^##^p < 0.01 **(C)** DHR-labeled *Mtb* (^DHR^*Mtb*) were incubated with 5 × 10^5^ PMN at a 10:1 ratio for 90 min. Results are expressed as a mean ± SE (n = 20). Ra vs. Ra + serum ^##^p < 0.01; M vs. M + serum ^##^p < 0.01; Ra vs. M ^φ^p < 0.01 **(D)** PMN were pretreated with DHR and incubated with 1:2 *Mtb*:PMN ratio for 90 min. The α-glucans of clinical strains were eliminated (Ra e, M e) or not (Ra, M) as described in Methods. Results are expressed as a mean ± SE (n = 15); control (−−) vs. Ra ***p < 0.001; Ra vs. Ra e: ^###^p < 0.001. In all cases emission of oxidized DHR was evaluated by flow cytometry **(E)** Apoptosis was measured in 18-hr cultured PMN in the same conditions described in **(A)**. Results are expressed as percentage of AV-FITC positive cells (% PMN AV + ± SE); control (−−) vs. Ra **p < 0.01; Ra vs. Ra e: ^###^p < 0.001.

In order to evaluate whether ROS production in PMN was dependent on bacterial phagocytosis, PMN were incubated with 10 μM of cytochalasin D (CytD), an inhibitor of actin polymerization, before the addition of *Mtb*. As can be seen in Figure 6A and B (1:2 and 10:1 *Mtb*:PMN ratio, respectively), the induction of ROS was significantly reduced on CytD-treated PMN and closely resembled the non-stimulated control, suggesting that Ra-induced ROS production requires the phagocytosis of the bacteria. Notably, when ROS was induced by DHR-labeled *Mtb* (oxidized DHR exclusively comes from ingested bacteria, ^DHR^*Mtb*), CD11b blockade did not affect ROS production, and more over, opsonized bacteria showed a reduced capacity to induce ROS (Figure 6C). These results together support the motion that ROS production is mainly induced by phagocytosis of non-opsonized bacteria through a receptor different from CD11b. Considering that the α-glucans present in the bacteria cell wall participate in the phagocytosis of *Mtb*, we evaluated the role of α-glucans in ROS production. Figure 6D shows that removing α-glucans by enzymatic digestion with 1,4-α-glycosidase provoked a reduction in ROS to basal levels, suggesting that α-glucans participate in the oxidative process. Accordingly, the elimination of α-glucans abolished the ability to induce PMN apoptosis (Figure 6E).

### α-glucans did not participate in GM1 expression or IL-8 secretion by PMN

**Figure 7:**
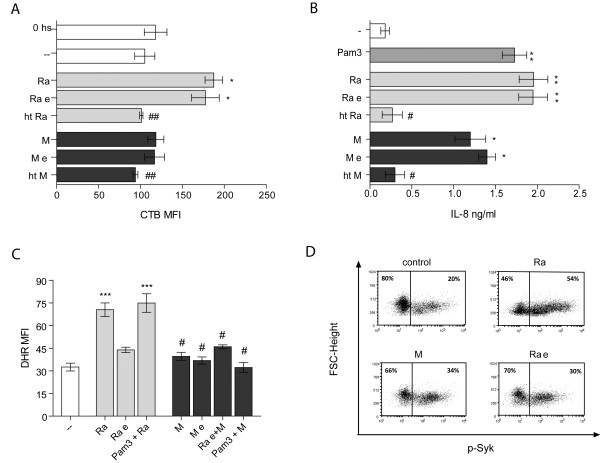
α**-glucans are not involved in the expression of GM1, or in the secretion of IL-8. (A)** PMN were cultured with Ra and M strains, enzimatically treated *Mtb* (Ra e, M e) or heat killed *Mtb* (ht Ra, ht M), in a 2:1 *Mtb*:PMN ratio. Thereafter cells were labeled with CTB-FITC and fluorescence evaluated by flow cytometry. Results are expressed as a mean ± SE (n = 12); control (−−) vs. Ra and Ra e: *p < 0.05; Ra and Ra e vs. ht Ra ^##^p < 0.01; M and M e vs. ht M: ^##^p < 0.01. **(B)** PMN were cultured for 18 hr at 1:2 *Mtb*:PMN ratio. Pam3Cys was used as control. IL-8 was measured in supernatant employing ELISA kits as described in Methods. Results are expressed as mean ± SE (n = 10); control (−−) vs. Ra, Ra e and Pam3Cys: **p < 0.01, control (−−) vs. M and M e: *p < 0.05; Ra and Ra e vs. ht Ra ^#^p < 0.05; M and M e vs. ht M: ^#^p < 0.05. **(C)** PMN incubated with DHR were cultured with enzimatically treated Ra (Ra e) or Pam3Cys and thereafter cells were challenged with M for additional 60 min (1:2 *Mtb*:PMN ratio). Emission of oxidized DHR was evaluated by flow cytometry. Results are expressed as a mean ± SE (n = 12); control vs. Ra or Pam3Cys + Ra: ***p < 0.001; Ra or Pam3Cys + Ra vs. Ra e, M, M e, Ra e + M or Pam3Cys + M: ^#^p < 0.05. **(D)** Percentage of cells that express the activated form of Syk in PMN incubated with Ra, M or enzimatically treated Ra (Ra e) clinical strains. One representative experiment of 10 is depicted.

Given that ROS production depends on α-glucans in the bacteria, but also depends on lipid rafts coalescence, we wondered if the α-glucans would be participating in the coalescence of lipid rafts. Next, we evaluated the ability to induce expression of GM1 by enzymatically treated bacteria and we found that the cell wall structure must be intact, because heated *Mtb* lost its ability to induce expression of GM1, however, removal the α-glucans had no effect, indicating that this component of the capsule is not involved in the coalescence of lipid raft, despite being involved in the production of ROS (Figure 7A). Moreover, clinical strains differentially induce IL-8 secretion by PMN as determined in the supernatant of 18-hour culture of PMN stimulated by Ra and M. The TLR2 agonist, Pam3Cys, was also employed as stimulus because IL-8 production is mediated by TLR in PMN [40]. As shown in Figure 7B, the secretion of IL-8 induced by strain Ra reached comparable levels to Pam3Cys, while strain M induced a lower effect, even if significant. In addition, the removal of the α-glucans did not alter *Mtb*-induced IL-8 secretion, indicating that α-glucans do not mediate the production of this cytokine. Besides, while heated bacteria abolish lipid raft coalescence and IL8 secretion, as this treatment alter recognition of TLR2 ligands, the enzyme treatment did not affect these surface structures.Considering that M did not induce GM1 expression, we wondered if their inability to induce ROS could be due to a failure in the induction of lipid rafts, in the interaction through the α-glucans or both. To assess this, PMN were first incubated with enzymatically-treated Ra (Ra e), which retains the ability to induce lipid rafts but loses the ability to generate ROS, and then M was added. In the same manner, other experiments were carried out employing the TLR2 agonist, Pam3Cys. The results show that even in these conditions, M was unable to induce ROS (Figure 7C) suggesting that both events are compromised: TLR2-dependent lipid raft coalescence and the presence of α-glucans in the cell wall. Finally, signaling via Syk might involve α-glucans, given that their removal significantly reduced Syk activation (Figure 7D).

## Discussion

The virulence of a certain *Mtb* genotype can be defined as its ability to cause active disease in humans. Thus, the level of clustering, revealed by molecular genotyping techniques, can be regarded as a measurable parameter of virulence [41]. Herein, we focused on two MDR-clinical strains representative of two families that nowadays show prevalent geographic niches [17]. While the genomic differences between these genotypes may be slight, they may lead to remarkable phenotypic differences among strains that influence epidemiologic and clinical outcomes of the disease [42].

Although mutations that confer drug resistance can imply a fitness cost, some MDR *Mtb* genotypes are able to overcome this disadvantage and are as virulent as fully drug-sensitive genotypes [43]. Unlike other pathogens, *Mtb* lacks typical virulence factors such as toxins; therefore, epidemiologic fitness of a strain can be influenced by a range of factors, for instance, the genetic background of host and pathogen, host–pathogen interactions, and the environment [44,45]. Thus, the accumulation of drug resistance-conferring mutations would be expected to reduce the epidemiological fitness of the strain M, but they have prevailed for over 15 years. Compensatory evolution restoring in vivo fitness, as well as social and behavior factors, may have played a role in the epidemiological persistence of strain M.

The intracellular replication of the pathogen and its spread from the lungs to other sites occur before the development of adaptive immune responses. Thus, innate immune mechanisms play an important role during the early stages of *Mtb* infection. In this context, PMN are the first cells to arrive at the site of infection and play a significant protective role in the elimination of invading pathogens through ROS production [20] or by limiting inflammation by succumbing to apoptosis [27]. Therefore, inhibition of ROS would increase not only the survival of the bacteria but also of PMN, which represents a remarkable *Mtb* trick to establish an abiding protected niche where bacteria can avoid elimination by the immune system [46,47].

We have previously demonstrated that clinical strains belonging to the LAM family have a strong ability to induce PMN apoptosis that depends on their capability to generate ROS production. However, clinical strains from Haarlem family induce less PMN apoptosis and ROS and particularly, strain M is unable to induce apoptosis due to a failure to stimulate ROS production and the induction of anti-apoptotic pathways [28].

Herein, we show that ROS generation and apoptosis induced by the MDR strain LAM Ra are dependent on dectin-1 and TLR2 receptors with the subsequent activation of p38 MAPK and Syk. In this context, the involvement of TLR2 and p38 MAPK pathway has also been described in ROS production induced with PPD in MØ [12]. Furthermore, these differences could not be ascribed to a modulation of TLR2 or dectin-1 expression, which is in contrast with that observed in non-phagocytic cells [48]. Moreover, Ra-induced Syk activation involves dectin-1 receptor, as described for zymosan [14,15], and *M. abscessus*[37]. Moreover, TLR2/dectin-1 cooperation has been described to induce the production of ROS, together with the rapid and sustained phosphorylation of p38 MAPK and Syk [37], in Mo and PMN [26,49].

It has been recognized that a variety of pathogens interact with plasma membrane lipid rafts which are involved in the attachment, cell entry, and intracellular survival of several microorganisms such as *Brucella suis*[50], *Chlamydia trachomatis*[51], and *M. bovis*[52]. In addition, components of the NADPH oxidase localize in membrane lipid rafts which are signaling platforms enriched in cholesterol [53], which mediates the efficiency of oxidase coupling to the receptor in PMN. In this context, we demonstrated that lipid rafts participate in Ra-induced ROS production and in this way rafts formation depends on TLR2. As lipid rafts also depended on ROS, we propose that some level of ROS generated by TLR2 signaling is necessary to induce the lipid rafts coalescence. Accordingly, a translocation of TLR2 to lipid rafts associated with ROS production has been demonstrated with *Mtb* 19 kDa lipoprotein [37].

Herein, we show that phagocytosis of *Mtb* is necessary to trigger ROS production. In addition, complement-mediated phagocytosis was similar in both strains, whereas M was less efficient than Ra when not opsonized [28]. The carboxyl-terminal end of the extracellular portion of the CD11b α-chain contains a lectin like C-domain involved in binding to oligosaccharides [38] including binding to mycobacterial polysaccharides [39]. Thus, we considered CD11b as a receptor which differentially binds these two strains. However, while the CD11b blockade reduces the non-opsonic phagocytosis of Ra, M remains significantly lower (Figure 5B) suggesting that another receptor may be involved. Moreover, when oxidized DHR comes from ingested bacteria, the blockade of CD11b does not affect ROS production, whereas opsonized bacteria show a reduced capability to induce ROS (Figure 6C). These results support the notion that ROS production is mainly induced by phagocytosis of non-opsonized bacteria through a different receptor from CD11b. In line with this, the enzymatic digestion of α-glucans prevents phagocytosis of non-opsonized bacteria, suggesting a role for α-glucans in phagocytosis, Syk activation and ROS production.

Little is known about how dectin-1-dependent inflammatory signaling is regulated during mycobacterial infection. The involvement of dectin-1 as a receptor to α-glucans is unlikely because it is associated with the recognition of β-glucans [13]. However, it has also been implicated in antimycobacterial immunity, though the ligand/s has not yet been identified [26,37,54]. Furthermore, dectin-1 also differentially recognizes glucans based on structural factors that include polymer length and side-chain branching [55,56] making it highly specific for glucans that have a (1 → 3)-β-D-glucopyranosyl backbone. Sugar recognition through C-type lectins has been found to be altered depending on the degree of saccharide acylation [57]. Therefore, the differences in the production of ROS between Ra and M strains may be due to its ability to enter the PMN and signal through Syk.

## Conclusions

In the present study, we investigated the mechanisms governing *Mtb*-induced ROS in PMN. Our results demonstrate that ROS is generated during the phagocytosis of non-opsonized mycobacteria through α-glucans and with the participation of dectin-1 and TLR2 involving p38 MAPK and Syk activation. Clinical strain M lacks the ability to induce ROS because of: 1) a reduced phagocytosis and 2) reduced lipid raft coalescence, a mechanism that would be beneficial for intracellular *Mtb* survival. Confirmation of this hypothesis awaits capsular characterization, which we are currently evaluating.

## Abbreviations

AV: Annexin V; APC: Antigen presenting cells; CR: Complement receptors; CTB: Cholera toxin B subunit; DHR: Dihydrorhodamine 123; DPI: Diphenyleneiodium; GM1: GM1 ganglioside; IL: Interleuquina; MAPK: Mitogen-Activated Protein Kinases; MβC: Methyl-β-cyclodextrin; MDR: Multidrug-resistant; Mtb: Mycobacterium tuberculosis; NADPH: Nicotinamide Adenine Dinucleotide Phosphate; PMN: Polymorphonuclear cells; PPD: Purified protein derivative; TB: Tuberculosis; TLR: Toll-like receptors; ROS: Radical Oxygen Species.

## Competing interests

The authors declare that they have no competing interests.

## Authors’ contributions

MMR conceived the study, carried out experimental procedures, and drafted the manuscript. LIB participated in performing assays and in revising the manuscript. BL and VR tested drug susceptibility of *Mtb* strains and genotyped by IS*6110* DNA fingerprinting and spoligotyping. MA conceived the study, revised experimental procedures and manuscript. MCS and LB helped to draft and to revise the manuscript. All authors read and approved the final version of the manuscript.

## Pre-publication history

The pre-publication history for this paper can be accessed here:

http://www.biomedcentral.com/1471-2334/14/262/prepub
